# Effects of feeding strategies during lay on broiler breeder production performance, eggshell quality, incubation traits, and behavior

**DOI:** 10.1016/j.psj.2023.102630

**Published:** 2023-03-08

**Authors:** R.A. van Emous

**Affiliations:** Wageningen Livestock Research, De Elst 1, NL-6708 WD Wageningen, the Netherlands

**Keywords:** broiler breeder, twice a day feeding, split-feeding, eggshell quality, behavior

## Abstract

An experiment was conducted to investigate the effects of a standard diet twice a day or split-feeding in broiler breeders on production performance, eggshell quality, incubation traits, and behavior. A total of 720 Ross 308 female breeders (45 weeks of age [**WOA**]) and 24 males (25 WOA) were randomly placed in 24 floor pens. The birds followed 3 feeding strategies: 1) Standard breeder diet fed once a day (100% at 0730 h) (**CON**), 2) Standard breeder diet fed twice a day (50% at 0730 h and 50% at 1600 h) (**TAD**), and 3) Split-feeding fed twice a day, with a morning (0730 h) and afternoon (1600 h) diet (**SF**). The morning diet contained more energy, protein, and phosphorus (**P**) and less calcium (**Ca**) than the control and afternoon diets. The afternoon diet had lower energy, protein, and P and higher Ca content than the control and morning diets. The TAD and SF birds tended to have a lower water intake (*P* = 0.055) and water-to-feed ratio (*P* = 0.054) compared to the CON birds. A 2.1% points higher hen-day egg production was found for the SF birds compared to the CON birds (*P* = 0.063), whereas the TAD birds did not differ from the other treatments. No differences were found for egg weight, eggshell quality, fertility, embryonic mortality, or average feather cover. A tendency toward a higher albumen percentage (*P* = 0.060) and lower yolk percentage (*P* = 0.069) was found for the TAD birds compared to the SF birds. The albumen-to-yolk ratio was higher (*P* = 0.022) for the TAD birds than for the CON and SF birds. Due to the twice-a-day feed distribution, the TAD and SF birds showed considerably different behavioral patterns than the de CON birds. In conclusion, twice-a-day feeding decreases the water intake and water-to-feed ratio, whereas split-feeding tended to an improved egg production in broiler breeders. However, no effects were observed on eggshell quality and incubation traits, whereas the behavioral patterns of the birds fed twice a day differed considerably with potential better welfare.

## INTRODUCTION

Broiler breeders are kept to produce as many first class hatching eggs as possible. A reduced eggshell quality, however, has a negative effect on the number of first class hatching eggs, hatchability, and chick quality (Leeson and Summers, 2005). Eggshell quality in broiler breeders usually decreases at the end of the production period; therefore, breeders are fed diets with an increased dietary calcium (**Ca**) level from approximately 40 weeks of age (**WOA**). Moreover, they are often fed an additional Ca source (e.g., oyster shell or large limestone) to support shell quality (Leeson and Summers, 2005).

Broiler breeders are fed a single portion of feed in the morning, which may impair the availability of nutrients at the correct time of day (e.g., Cave, 1981). In particular, the availability of Ca during the evening and night is crucial for eggshell formation (Farmer et al., 1983a,b). Formation of the eggshell starts in the afternoon and/or early evening, which is hours after the daily portion of feed in the morning (Backhouse and Gous, 2005). Broiler breeders with a single amount of feed in the morning show a feed clean-up time within 2 to 4 h (Roland and Farmer, 1984; Backhouse and Gous, 2005). Research on broiler breeders showed that more than half of the absorbed Ca had disappeared from the crop within 4 h of ingesting the feed (feeding from 0700 am onwards) (Farmer et al., 1983b). The latter also showed that broiler breeders absorb about 25% of the absorbed Ca in the intestines in the first 4 h. These birds were fed at 0700 h and absorbed only a very low percentage of Ca from 1100 h until 2300 h (Farmer et al., 1983b). The Ca required for the eggshell is then released from the Ca stores in the bone, which can result in reduced bone quality and leg problems. Feeding broiler breeders later in the day makes Ca more available at the time of eggshell formation (Farmer et al., 1983a). This results in better Ca utilization (Farmer et al., 1983b; Roland and Farmer, 1984), usually reflected in an increase in egg specific gravity, shell weight, and shell thickness (Backhouse and Gous, 2006). Bootwalla et al. (1983), Farmer et al. (1983a), Backhouse and Gous (2005), and Londero et al. (2015) showed that birds fed only in the afternoon had the best eggshell quality. However, experiments performed by Wilson and Keeling (1991), Samara et al. (1996), Spradley et al. (2008), and van Emous and Mens (2021) showed no effect of feeding time on eggshell quality.

More recently, a novel feeding strategy in layer hens was applied in which birds were fed via a split-feeding (specific morning and afternoon diet) program to facilitate egg and eggshell formation (de Los Mozos, 2014; Molnár et al., 2018). The specific morning diet contained more energy, protein, and phosphorus (**P**) and less Ca than a standard breeder diet, where the afternoon diet had a lower energy, protein, and P and higher Ca content than a standard breeder diet. de Los Mozos (2014) showed that split-feeding in laying hens between 95 and 98 WOA improved eggshell quality. The percentage of cracked eggs was 30% lower, eggshell weight was 1.3% higher, eggshell thickness was 1.3% thicker, and eggshell weight per surface (mg/cm^2^) was 9% higher. van Krimpen et al. (2018) further showed that the use of split-feeding in organic laying hens resulted in a lower P excretion without negative effects on egg production and eggshell quality. Research with split-feeding in broiler breeders has shown that this can result in higher egg and chick production (Anonymous, 2021; van Emous and Mens, 2021). In the experiment by van Emous and Mens (2021), broiler breeders between 51 and 55 WOA fed the split-feeding strategy showed a tendency toward 3.2% points higher egg production. Moreover, in an on-farm study at a large Spanish integration with more than 120,000 broiler breeders, 1.9 higher number in chick production was observed (Anonymous, 2021). Until now, only 1 short (pilot) experiment and some on-farm experiments have been carried out on the effects of applying split-feeding in broiler breeders. Therefore, the present experiment was conducted to determine the effects of providing a standard diet twice a day or split-feeding in broiler breeders on production performance, eggshell quality, incubation traits, and behavior.

## MATERIALS AND METHODS

### Experimental Design

The trial consisted of 3 feeding strategies, and each treatment had 8 replicates. The control treatment breeders (**CON**) received the standard breeder diet once a day at 0730 h, whereas the twice-a-day treatment breeders (**TAD**) received the standard breeder diet at 0730 h and 1600 h at a ratio of 50:50. For the split-feeding treatment breeders (**SF**), specific morning (at 0730 h) and afternoon (1600 h) diets were used (Table 1). The morning diet contained more energy, crude protein, and P and less Ca compared to the control and afternoon diets. The afternoon diet contained less energy, crude protein, and P and more Ca compared to the control and morning diets. To avoid confounding effects, the diets were formulated so that the average nutritional value of the morning and afternoon diets was comparable to that of the control diet. In addition, the morning diet consisted of 80% fine Ca source (chalk) and 20% coarse Ca source (limestone), whereas the afternoon feed consisted of 80% coarse and 20% fine Ca source. The fine Ca source in the morning diet was used to support medullary bone formation, and the coarse Ca source in the afternoon diet was used to allow a more constant Ca release to the plasma pool, thus facilitating eggshell formation during the night (Molnár et al., 2018; van Emous and Mens, 2021).

**Table 1:** Dietary ingredients and analyzed and calculated nutrients of the pullet diets (g/kg, as-fed basis).

Item	Control diet	Split feeding morning diet	Split feeding afternoon diet
Ingredient			
Maize	376.2	376.2	376.2
Wheat	249.9	275.8	230.3
Wheat middling	45.8	12.4	60.0
Rapeseed meal	49.5	49.5	49.5
Sunflower meal	79.2	80.2	80.2
Soybean meal	49.9	63.0	33.1
Peas	15.0	30.0	15.0
Soya oil	22.9	24.1	19.9
Salm oil	4.0	4.0	4.0
Salcurb dry K2	5.0	5.0	5.0
Limestone	65.5	11.3	84.8
Chalk	15.0	45.3	21.0
Monocalcium phosphate	3.2	4.3	2.2
Salt	1.4	1.4	1.4
Sodium carbonate	3.4	3.5	3.4
Premix lay^1^	4.0	4.0	4.0
DL-Methionine	1.1	1.2	0.9
L-Lysine	0.9	0.8	0.9
L-Threonine	0.6	0.7	0.5
Choline-chloride	1.5	1.5	1.5
Water	6.1	6.0	6.1
Calculated content^2^			
AMEn (kcal/kg)	2,800	2,900	2,700
Crude ash	111.3	88.4	135.1
Crude protein	139.0	146.3	131.3
Crude fat	48.0	48.7	45.0
Crude fiber	41.2	40.0	41.7
Starch	409.0	425.6	399.6
Dig. Lys	5.30	5.70	4.90
Dig. Met+Cys	5.48	5.82	5.12
Dig. Thr	4.51	4.84	4.16
Dig. Trp	1.33	1.40	1.23
C18:2 linolenic acid	23.56	24.04	21.97
Sodium	1.60	1.60	1.60
Potassium	5.99	6.12	5.75
Chloride	1.90	1.90	1.90
dEB (mEq/kg)	169.4	172.7	163.2
Calcium	33.47	24.23	43.10
Total phosphorus	4.46	4.61	4.25
Available phosphorus	3.10	3.30	2.90
Analyzed content			
DM	879.0	878.0	883.0
Crude ash	98.0	86.0	125.0
Crude protein	142.0	144.0	130.0
Crude fat	52.0	54.0	49.0
Crude fiber	45.0	45.0	42.0
Starch	400.0	396.0	397.0
Total calcium	31.7	25.1	41.1
Total phosphorus	4.13	4.58	3.97

1 Provided per kilogram of complete diet: vitamin A, 10,050 IU; vitamin B1, 3.0 mg; vitamin B2, 12.1 mg; vitamin B3, 48.2 mg; vitamin B4, 281.4 mg; vitamin B5, 15.1 mg; vitamin B6, 4.0 mg; vitamin B9/B11, 1.6 mg; vitamin B12, 0.03 mg; vitamin D3, 2,513 IU; vitamin E, 40.2 mg; vitamin H, 0.2 mg; vitamin K3, 3.0 mg; iron, 64.3 mg; copper, 5.0 mg; manganese, 30.2 mg; zinc, 30.2 mg; iodine, 1.5 mg; selenium, 0.4 mg.

2 CVB matrix values (CVB, 2016) were used for diet formulation.

### Breeders, Housing, and Management

The experiment was conducted between 45 and 65 WOA with 720 Ross 308 female broiler breeders. Before the experiment, a pre-experimental period (44–45 WOA) was applied to acclimate the birds to the new environment. Birds originated from a commercial broiler breeder farm and were randomly divided into 24 pens in 2 identical climate-controlled rooms. Two additional pens were available for 30 spare birds to replace birds during the first week of the experimental pens due to mortality and grading (too light, too heavy, not laying, and injured). During the pre-experimental period, all birds received a standard breeder diet. When females were 45 WOA, young males (Ranger Gold, 21 WOA) originating from a commercial rearing farm were housed in 2 additional pens until sexual maturity (25 WOA). Per pen, 1 sexually mature male was placed at 49 WOA and 1 at 50 WOA (female age). Due to aggressive behavior between the males, however, 1 male was removed after 2 d. As a result, the study continued with 1 male per pen (24 in total; male to female ratio of 1 to 30). Initially stocking density was 6.2 birds per m^2^ or 1,613 cm^2^/bird (30 females and 1 male per pen).

The pens were each 2.5 × 2.0 m (5 m^2^) and contained an elevated floor (1.5 × 1.0 m; 1.5 m^2^), 2 plastic perches (total length 4 m), and a litter area (3.5 m^2^) with wood shavings (2.0 kg/m^2^) as bedding material. Outside each pen, adjacent to the slats, 1 nest box (88 × 36 cm) was placed. Females were manually fed in 2 feeding troughs (total 3.7 m length; 12.3 cm feeding space per female) containing a grill to prevent male access to the feed. The males were manually fed with a male feeding pan, which was placed above the litter area at a minimum height of 50 cm to prevent female access. Water was available ad libitum during the light period via 7 nipple drinkers with drip cups positioned above the slatted floor. During the experiment, the birds of the different feeding strategies were maintained on the same target body weight (**BW**). Feed allocation was adjusted to the predetermined body growth curve and egg production (Aviagen-EPI, 2017). Males were fed once a day (0730 h) a commercial male diet (2,600 kcal/kg AMEn; 13.0% CP; 0.45% dig. Lys; 0.5% dig. M+C; 1.0% Ca; 0.3% aP). Room temperature was maintained at 20°C, and the photoperiod was 14L:10D (40 lx), with the lights on from 0700 to 2100 h. Birds were visually observed twice a day to check their health. This study was approved by the Dutch Central Authority for Scientific Procedures on Animals (CCD) and is registered under application number AVD4010020185007.

### Observations

*Diet analysis:* The experimental diets were formulated and produced by ABZ Diervoeding, Leusden, the Netherlands. Diets were analyzed for dry matter, crude ash (ISO 5984), crude protein (ISO 5983), crude fat (ISO 6492), crude fiber (ISO 6865), starch (NEN-EN-ISO 15914), Ca (ISO 6869), and phosphorous (ISO6941). All analyses were performed in duplicate and carried out by NutriControl, Veghel, the Netherlands.

*Body weight:* To monitor BW and BW gain, 10 females (as a group) and the lone male per pen were weighed weekly in the morning before feeding.

*Feed allocation and water intake:* Feed allocation was adjusted for mortality, and the different diets according to the predetermined body growth curve and egg production. Water intake was recorded daily by recording water level of the container per pen and calculated per week for the entire experimental period.

*Production performance:* All eggs per pen were collected daily, graded, and recorded. The total number of settable (above 50 g), small (under 50 g), double yolk, abnormal eggshell, dirty, and floor eggs was calculated per week and for the total experimental period on a pen basis. On a weekly basis, on the same day of the week, all hatching eggs (settable and small) were weighed. The average egg weight for the entire experimental period was calculated.

*Incubation Traits:* Incubation traits were measured at 52, 59, and 65 WOA. Per pen, 50 eggs (collected from 3 d of production) were placed in an incubator after a 5- to 7-d storage period (16°C–18°C and 50–60% RH). On d 7 of incubation, all eggs were opened to identify unfertilized eggs and total embryonic mortality.

### Eggshell Quality

*Breaking strength:* At 45, 50, 55, 60, and 65 WOA, the maximum breaking strength of the eggshell of 15 first-grade eggs per pen was determined using the Futura Egg Shell Tester Ver. 2 (FEST) (Bröring Informationstechnology, Lohne, Germany). Breaking strength was measured in Newtons (N) and indicated the moment at which the eggshell broke due to the compressive force exerted on the shell by the device. Possible values that could be measured ranged from 0 to 75 N. Breaking strength determination was determined by candling whether the eggshell showed any hairline cracks or other shell damage. Before the start of the test, the FEST was calibrated using compression weights of 500 and 1,000 g.

*Eggshell thickness:* At 45, 50, 55, 60, and 65 WOA, the eggshell thickness of 10 first grade hatching eggs was measured. Eggshell thickness was determined at 3 locations of the egg: top, middle, and bottom (van Krimpen et al., 2018). Pieces of shells of a few square millimeters of surface were used so that the curvature of the eggshell was as small as possible, and the inner shell membrane of the eggshell was removed. Eggshell thickness was determined with an eggshell thickness gauge (Bröring Informationstechnology, Lohne, Germany), ranging from 0.001 to 3.500 mm. Eggshell thickness was determined in mm (2 decimals).

*Albumen-to-yolk ratio and eggshell weight:* At 45, 50, 55, 60, and 65 WOA, the albumen-to-yolk ratio and eggshell weight of 10 first-grade eggs per pen were determined. The eggs were first weighed fresh and then boiled for 10 min and weighed again; thereafter, the yolk and albumen were separated and weighed. The eggshell weight was determined immediately after boiling and after drying at room temperature for 24 h. The dry matter of the eggshell was calculated.

*Specific gravity:* At 45, 50, 55, 60, and 65 WOA, 15 first-grade hatching eggs were used to examine specific gravity using the saline solution floating method (Montenegro et al., 2019). The collected eggs were fresh, up to 36 h old, and stored at about 15°C. Five containers were filled with water and placed in the measuring chamber 24 h before specific gravity determination. On the measuring day, salt (NaCl) was added such that the density in the 5 containers was 1.070, 1.075, 1.080, 1.085, and 1.090 g/cm^3^ (determined with a hydrometer). Individual eggs were placed in containers from lower to higher sodium chloride solution concentrations. The concentration of sodium chloride solution was the specific gravity of the egg when the egg was suspended. Afterwards, the eggs were dried and weighed individually.

*Feather cover:* At 45, 50, 55, 60, and 65 WOA, the feather cover of 5 random birds per pen was scored according to the method described by Bilcik and Keeling (1999). Scores, varying from 0 (intact feathers) to 5 (completely denuded area), were given for each of the 7 body parts (neck, breast, belly, back, wings, tail, and legs). The average of these 7 scores was also used for analysis.

*Behavior:* The home pen behavior of the birds was observed by live scan sampling of each pen at 45, 50, 55, 60, and 65 WOA. Behavior observations were performed by 2 pretrained people during the observation day, consisting of 12 observation sessions throughout the light period. The first observation session started at 0700 h and was 30 min before the first feeding time, and the sessions were repeated each hour until the last one at 1800 h. Before each observation session, 5 min of habituation time per compartment was observed, and the observers switched rooms between observation sessions. Behavior was scored by counting the birds performing different behaviors according to the ethogram previously described by van Emous et al. (2015) (Table 2). Feeding and drinking were recorded only when feed and water were available. During feed availability, object pecking was defined as pecking at the pen or equipment, and when feed troughs were empty, pecking at the feeder was also scored as object pecking.

**Table 2:** Ethogram of behavioral observations (based on van Emous et al., 2015).

Behavior	Definition
Eating	Pecking at feed at the feeding troughs
Drinking	Pecking at water at the nipple drinkers
Standing	Standing without performing other behavior
Sitting	Sitting without performing other behavior
Walking	Walking or running without performing other behavior
Foraging	Pecking and/or scratching the litter
Comfort	All comfort behavior like, preening, auto pecking, nibbling, stroking, wing flapping, and stretching
Dustbathing	Dustbathing behavior
Object pecking	Stereotypic pecking at parts of the pen, wall, empty feeding pans, or empty nipple drinkers
Bird pecking	All pecking at other birds

### Statistical Analysis

The data were analyzed using Genstat statistical software (Genstat, 2018). Response variables with regard to production performance were analyzed using ANOVA according to the following model: Y_ijk_ = μ + R_i_ + FS_j_ + OS_k_ + ε_ijk,_ where Y_ijk_ is the response variable, μ is the overall mean, R_i_ is the random effect of room (i = 1, 2), FS_j_ is the effect of feeding strategy (CON, TAD, SF; j = 1..3), OS_k_ is the effect of observation term session (k = 1..12), and ε_ijk_ is the residual error term. The statistical model for incubation traits, eggshell quality, and behavior included age as a fixed effect. Parameters were tested for normal distribution before analysis. After inspection of the diagnostic plots of the residuals, the behavioral variables were analyzed with a logistic regression model. Pen was treated as the experimental unit. A statistically significant difference was considered at *P* ≤ 0.05, with 0.05 < *P* ≤ 0.10 considered a tendency.

## RESULTS AND DISCUSSION

### Diet Composition

The analyzed dry matter content of the diets was, on average, 1.1% lower than the calculated content (Table 1). The analyzed crude protein content of the control diet was 2.2% higher than the calculated content, whereas the CP content was 1.6 and 1.0% lower for the morning and afternoon diets, respectively, than the calculated content. The analyzed crude fat content was 8.3, 10.9, and 8.9% higher for the control, morning, and afternoon, respectively, than the calculated content. The analyzed Ca content was 5.3, and 4.6% lower for the control and afternoon diets, respectively, whereas the analyzed Ca content was 3.6% higher for the morning diet compared to the calculated content. The desired contrast in Ca levels between the different diets was, however, still present. The analyzed P content was 7.4, 0.7, and 6.6% lower for the control, morning, and afternoon diets, respectively, compared to the analyzed content.

### Body Weight

To prevent confounding effects during experiments, broiler breeders were fed to the same bodyweight targets, resulting in no differences in BW between the females and males (data not shown).

### Feed Allocation, Water Intake, and Water to Feed Ratio

The average feed allocation for the different feeding strategies was equal for females (162.6 g/b/d) and males (168.8 g/b/d) during the experimental period. A tendency for a lower water intake (*P* = 0.055) and water-to-feed ratio (*P* = 0.054) was found for birds fed twice a day (TAD and SF) (Table 3). This is in contrast to the previous study by van Emous and Mens (2021) with twice-a-day feeding and split-feeding, who did not find an effect on water intake and water-to-feed ratio. The lower water intake in the present study was substantiated by the behavioral observations, where the birds fed twice a day (TAD and SF) showed a tendency (7.8% and 8.2% vs. 8.9%; *P* = 0.080) toward less drinking behavior compared to the CON birds (Table 7). The reason for the difference in water intake is not well understood, but it is probably caused by dividing the total feed amount into 2 portions during the day. During behavioral observations, the birds fed twice a day were calmer and showed more resting (standing and sitting) between the 2 feeding times what is an indication of less boredom and improved satiety (Hocking and Maxwell, 1996).

**Table 3:** Effects of feeding strategies on production performance.

Feeding strategy^1^	Water intake (ml/b/d)	Water to feed ratio	Hen-day egg production (%)	Hatching eggs (#)	Abnormal shell eggs (%)^2^	Dirty eggs (%)	Floor eggs (%)	Egg weight (g)	Mortality (%)
CON	278.6	1.71	72.4	63.8	1.58	4.03	6.1	66.3	7.6
TAD	265.3	1.63	74.2	63.1	1.30	4.61	8.9	66.8	10.4
SF	265.0	1.63	74.5	64.8	1.51	4.21	7.1	66.2	5.4
SEM	4.21	0.026	0.64	1.51	0.142	0.553	1.89	0.23	1.54
*P*-value	0.055	0.054	0.063	0.73	0.39	0.75	0.57	0.23	0.098

1 CON = control diet once a day; TAD = twice-a-day feeding: control diet twice a day; SF = split-feeding: special morning and afternoon diets.

2 Abnormal shell eggs = cracked, soft shell and shell less eggs.

### Production Performance

The SF birds tended to have a higher average hen-day egg production (%) throughout the experimental period compared to the CON birds (74.5% vs. 72.4%; *P* = 0.063; Table 3). The TAD birds (74.2%) did not differ from the SF and CON birds (Table 3). Higher egg production for SF birds is in agreement with research by van Emous and Mens (2021) and Anonymous (2021). van Emous and Mens (2021) found a tendency to a 3.2% points higher egg production between 51 and 55 WOA for split-feeding compared to the control breeders (once-a-day standard breeder diet). Anonymous (2021) found significantly higher egg production between 55 and 60 WOA, which resulted in more eggs and more chicks. In another study, in collaboration with a large Spanish integration in more than 120,000 parent stock, 1.9 higher number in chick production was found (Anonymous, 2021). Birds in the present study that received the twice-a-day feeding strategy showed no higher egg production than birds that received the control feeding strategy. This is in agreement with research by Cave (1981), Bootwalla et al. (1983), Samara et al. (1996), and Backhouse and Gous (2005), who also found no differences in egg production when using different feeding times and twice-a-day feeding. This is, however, in contrast to research by de Avila et al. (2003), Spradley et al. (2008), Taherkhani et al. (2010), Moradi et al. (2013), and Soltanmoradi et al. (2013), who found higher egg production when breeders were fed twice a day compared to breeders fed once a day. It is hypothesized that differences in the effects of twice-a-day feeding between studies on egg production are caused by differences in breeds, feeding schedules, and the length of the applied twice-a-day feeding period.

No differences were found for other production characteristics and egg weight (Table 3), which agrees with studies by Harms (1991) and Samara et al. (1996), who applied twice-a-day feeding. However, Cave (1981), Spradley et al. (2008), and Moradi et al. (2013) found higher egg weights when breeders were fed 2 or 3 times a day.

A tendency toward lower mortality was found for the SF birds compared to the TAD birds (5.4% vs. 10.4%; *P* = 0.098), whereas the CON birds (7.6%) did not differ from the other treatments (Table 3). No information regarding mortality was found in the literature in studies focusing on twice-a-day feeding and split-feeding. The reason for the differences in mortality is unclear.

### Incubation Traits

No effects of the different treatments were found on fertility and embryonic mortality (Table 4), which is in line with the previous studies of Spradley et al. (2008) and van Emous and Mens (2021). Soltanmoradi et al. (2013), however, found that feeding breeders twice a day resulted in higher fertility and hatchability. Before starting the experiments with twice-a-day feeding, it was hypothesized that this feeding strategy could potentially improve fertility due to increased bird (both females and males) activity during the last 3 to 4 h of the day. This part of the day is the optimal period for egg fertilization (Løvlie and Pizzari, 2007), and the majority of matings take place at the end of the day (Harris et al., 1980; Bilcik and Estevez, 2005). More activity and mixing of females and males during the last 3 to 4 h of the day can result in more mating behavior and higher fertility (van Emous, 2010).

**Table 4:** Effects of feeding strategies and age on fertility and embryonic mortality (EM).

Item^1^	Fertility (%)	Total EM (%)
Feeding strategy		
CON	94.3	1.3
TAD	93.8	1.0
SF	95.5	1.4
SEM	1.1	0.3
Age		
52 WOA	94.4	0.4^b^
59 WOA	96.0	1.0^ab^
65 WOA	93.2	2.3^a^
SEM	1.1	0.3
*P-value*		
Feeding	0.52	0.68
Age	0.18	<0.001
Feeding × Age	0.69	0.091

Abbreviation: WOA, weeks of age.

a-b Means within a column and within a source with no common superscript differ (*P ≤* 0.05).

1 CON = control diet once a day; TAD = twice-a-day feeding: control diet twice a day; SF = split-feeding: special morning and afternoon diets.

Embryonic mortality increased from 0.4 to 2.3% between 52 and 65 WOA (*P* < 0.001), which is in agreement with previous studies when breeder females aging (e.g., Fontana et al., 1992; Bramwell et al., 1996).

### Eggshell Quality

No treatment effects on breaking strength, eggshell thickness, eggshell weight (after cooking and after 24 h drying), DM eggshell, or specific gravity were observed (Table 5). These findings are in accordance with the studies of Samara et al. (1996), Backhouse and Gous (2005), Spradley et al. (2008), Londero et al. (2015, 2016), and van Emous and Mens (2021). In contrast to our findings, higher eggshell weights were found in breeders (Lewis and Perry, 1988; Soltanmoradi et al., 2013) and layers (de Los Mozos, 2014) when feeding twice-a-day. It has previously been postulated that the fineness of the Ca source in relation to the moment of provision is important for eggshell formation (Molnár et al., 2018). Ca in chalk is directly available to support Ca reabsorption to bone, and coarse limestone in the afternoon can support eggshell formation during the evening and night (Zhang and Coon, 1997; Leeson and Summers, 2005; Molnár et al., 2018). Therefore, in the present study, the morning diet contained 80% fine Ca source (chalk) and 20% coarse Ca source (coarse limestone), whereas the afternoon feed contained 80% coarse Ca source and 20% fine Ca source. Despite this improvement in diet composition, no effects were found on eggshell quality. The breaking strength of the eggs in the present study was still relatively high as birds aged (37.6 N at 65 WOA, Table 5). It is therefore hypothesized that, due to the high breaking strength in the current study, future experiments must be applied in older breeders when egg quality is really decreases.

**Table 5:** Effects of feeding strategies and age on breaking strength, eggshell thickness eggshell weight (after cooking and after 24 h drying), DM eggshell, albumen (%), yolk (%), albumen-to-yolk ratio, and specific gravity.

Item^1^	Breaking strength (Newton)	Eggshell thickness (mm)	Albumen (%)	Yolk (%)	Albumen-to-yolk ratio	Eggshell weight after cooking (g)	Eggshell weight after 24 h drying (g)	DM eggshell (%)	Specific gravity (g/cm^3^)
Feeding strategy									
CON	39.2	0.339	56.5	33.4	1.70^b^	6.44	5.78	89.9	1.080
TAD	39.4	0.338	56.7	33.2	1.72^a^	6.51	5.85	89.9	1.079
SF	39.9	0.342	56.3	33.5	1.69^b^	6.48	5.82	89.8	1.079
SEM	0.4	0.002	0.1	0.1	0.01	0.03	0.03	0.1	0.0002
Age									
45 WOA	39.9^b^	0.339	56.5^ab^	33.2^b^	1.71^b^	6.40^c^	5.72^c^	89.4^b^	1.080^a^
50 WOA	41.2^a^	0.342	56.8^a^	33.0^b^	1.73^ab^	6.50^ab^	5.86^ab^	90.1^a^	1.081^a^
55 WOA	40.1^ab^	0.339	56.8^a^	32.9^b^	1.74^a^	6.49abc	5.84^ab^	90.0^a^	1.080^a^
60 WOA	38.7^bc^	0.342	56.1^b^	33.8^a^	1.67^c^	6.55^a^	5.90^a^	90.1^a^	1.078^b^
65 WOA	37.6^c^	0.338	56.2^b^	34.0^a^	1.66^c^	6.44^bc^	5.78^bc^	89.8^a^	1.079^b^
SEM	0.4	0.002	0.1	0.1	0.01	0.04	0.03	0.1	0.0003
*P-value*									
Feeding	0.47	0.31	0.060	0.069	0.022	0.18	0.16	0.77	0.24
Age	<0.0001	0.25	<0.001	<0.001	<0.001	0.032	0.002	<0.001	<0.001
Feeding × Age	0.56	0.67	0.68	0.77	0.81	0.57	0.66	0.30	0.58

Abbreviation: WOA, weeks of age.

a-c Means within a column and within a source with no common superscript differ (*P ≤* 0.05).

1 CON = control diet once a day; TAD = twice-a-day feeding: control diet twice a day; SF = split-feeding: special morning and afternoon diets.

A tendency toward a higher albumen percentage (56.7% vs. 56.3%; *P* = 0.06) and a lower yolk percentage (33.2% vs. 33.5%; *P* = 0.069) for the TAD birds compared to the SF birds was found, whereas the CON birds did not differ. The albumen-to-yolk ratio was higher for the TAD birds than for the CON and SF birds (1.72 vs. 1.70 and 1.69; *P* = 0.022). Differences in egg composition can potentially affect the offspring because the yolk is the major energy source and both albumen and yolk are major protein sources for tissue synthesis for the developing embryo (Noble and Cocchi, 1990; Willems et al., 2014).

Different age-related effects for eggshell quality parameters were observed. Breaking strength was the highest between 45 and 55 WOA and decreased at 60 and 65 WOA. The eggshell weight (after cooking and after 24 h drying) was highest at 60 WOA and lowest at 45 WOA. The DM content of the eggshell was highest at 50 and 65 WOA and lowest at 45 WOA. The albumen percentage was highest between 45 and 55 WOA and lowest between 60 and 65 WOA; this was the opposite for the yolk percentage. The albumen-to-yolk ratio was highest between 45 and 55 WOA compared to 60 and 65 WOA.

### Feather Cover

The different treatments had no effect on the feather cover scores of the birds, except on the thigh (Table 6). The feather cover score increased (i.e., worse feather cover) until 60 WOA, but it improved (i.e., better feather cover) at 65 WOA. This improvement occurred because some birds molted and thus substituted their feather cover, resulting in better feather cover. Molting of breeders while aging is a biological phenomenon that originates from stress factors, such as diseases, loss of BW, climate problems, and essential nutrient deficiencies (Ellis, 2004). Birds stop producing eggs and replace their entire feather cover to start a new period of egg production. In the present study, the number of birds that molted was counted at 65 WOA (data not shown). No differences were observed in the percentage of molted birds between treatments; however, a high percentage of birds (approximately 10%) had molted, which explained the improved feather cover at an older age.

**Table 6:** Effects of feeding strategies and age on feather cover score of the different body parts and average.

Item^1^	Neck	Breast		Belly		Back		Wings		Tail		Legs		Average
Feeding strategy														
CON	1.17	3.44		3.34		3.21		2.45		2.88		2.91^b^		2.77
TAD	1.34	3.49		3.37		3.12		2.46		2.95		2.99^b^		2.82
SF	1.29	3.40		3.41		3.25		2.44		2.93		3.22^a^		2.85
SEM	0.06	0.07		0.07		0.096		0.414		0.07		0.07		0.05
Age														
45 WOA	1.07^c^	3.37^b^		2.88^c^		2.63^d^		2.37^b^		2.43^c^		2.43^d^		2.45^d^
50 WOA	1.30^ab^	3.07^c^		3.05^c^		3.00^c^		2.41^b^		2.90^b^		2.85^c^		2.65^c^
55 WOA	1.38^a^	3.56^ab^		3.52^b^		3.29^b^		2.38^b^		3.03^ab^		3.14^b^		2.90^b^
60 WOA	1.45^a^	3.59^c^		3.70^a^		3.72^a^		2.66^a^		3.13^a^		3.43^a^		3.10^a^
65 WOA	1.13^bc^	3.62^c^		3.70^a^		3.33^b^		2.44^b^		3.11^a^		3.33^a^		2.95^b^
SEM	0.08	0.08		0.07		0.09		0.41^b^		0.07		0.07		0.05
*P-value*														
Feeding	0.13	0.66		0.81		0.63		0.97		0.53		0.009		0.59
Age	<0.001	<0.001		<0.001		<0.001		0.002		<0.001		<0.001		<0.001
Feeding × Age	0.30	0.73		0.58		0.53		0.094		0.72		0.46		0.38

Abbreviation: WOA, weeks of age.

a-d Means within a column and within a source with no common superscript differ (*P ≤* 0.05).

1 CON = control diet once a day; TAD = twice-a-day feeding: control diet twice a day; SF = split-feeding: special morning and afternoon diets.

### Behavior

In contrast to the previous study by van Emous and Mens (2021), no differences in eating behavior were observed in the present study between twice- and once-a-day feeding (Table 7). The CON birds tended to spend more time drinking than the TAD birds (8.9% vs. 7.8%; *P* = 0.080), whereas the SF birds did not differ from the other treatments. This was in contrast to the previous experiment of van Emous and Mens (2021) in which no differences were found in drinking behavior. The birds fed twice a day (TAD and SF) spent more time standing than the CON birds (32.7% and 34.0% vs. 26.6%; *P* < 0.001), whereas no differences were found in the previous experiment (van Emous and Mens, 2021). There was a tendency to spend more time sitting for the CON birds compared to the SF birds (15.7% vs. 12.4%; *P* = 0.079), whereas the TAD birds did not differ from the other treatments. In the previous experiment, CON showed less sitting behavior (van Emous and Mens, 2021). The TAD birds showed a tendency to walk more than the CON birds (4.9% vs. 4.1%; *P* = 0.056), whereas the SF birds did not differ from the other treatments. In the previous experiment, there was no difference between the feeding strategies with regard to walking. The CON birds spent more time on foraging than the TAD and SF birds (13.7% vs. 11.5% and 11.1%; *P* < 0.001), which is consistent with the previous experiment (van Emous and Mens, 2021). There was a tendency to spend more time on dustbathing for the CON birds compared to the SF birds (1.7% vs. 1.1; *P* = 0.076), whereas the TAD birds did not differ from the other treatments. In the previous experiment, no differences were found between the feeding strategies for dustbathing behavior (van Emous and Mens, 2021). In the present experiment, no differences were found in object and bird pecking, whereas in the previous experiment, the breeders fed twice daily showed less object pecking. The differences in behavior between the 2 experiments may be due to differences in origin. The animals in the previous experiment were already present in pens from day-old chicks, whereas the birds in the current experiment originated from a commercial breeder farm.

**Table 7:** The effects of the different feeding strategies and age on behavior (% of time).

Item^1^	Eating	Drinking	Standing	Sitting	Walking	Foraging	Comfort	Dust-bathing	Object pecking	Bird pecking	Egg laying
Feeding strategy											
CON1x	22.0	8.9	26.6^b^	15.7	4.1	13.7^a^	5.4	1.7	0.5	0.9	0.4
CON2x	21.0	7.8	32.7^a^	13.4	4.9	11.5^b^	6.1	1.2	0.3	0.8	0.3
SP2x	21.2	8.2	34.0^a^	12.4	4.4	11.1^b^	5.7	1.1	0.5	1.0	0.3
SEM	1.2	0.5	1.5	1.4	0.2	0.9	0.3	0.2	0.1	0.1	0.1
Age											
45 WOA	24.9^a^	7.9^bc^	26.5^c^	18.0^a^	4.6^b^	11.1^bc^	4.5^b^	1.1	0.3^b^	0.9	0.2^b^
50 WOA	23.3^a^	8.3^b^	32.1^ab^	13.0^b^	5.3^a^	10.5^cd^	5.2^b^	1.0	0.3^b^	0.8	0.3^ab^
55 WOA	18.6^b^	7.2^c^	35.2^a^	10.3^c^	3.4^c^	16.9^a^	5.4^b^	1.4	0.3^b^	1.1	0.3^ab^
60 WOA	19.3^b^	9.2^a^	32.0a^b^	12.2^bc^	4.2^b^	12.6^b^	7.0^a^	1.5	0.7^a^	0.9	0.5^a^
65 WOA	21.1^ab^	8.9^ab^	29.7^bc^	15.9^a^	4.9^b^	9.3^d^	6.6^a^	1.7	0.5^ab^	1.1	0.4^a^
SEM	1.6	0.5	1.6	1.3	0.3	1.0	0.3	0.2	0.1	0.1	0.1
*P-value*											
Feeding	0.82	0.080	<0.001	0.079	0.056	<0.001	0.17	0.076	0.14	0.33	0.66
Age	0.022	<0.001	<0.001	<0.001	<0.001	<0.001	<0.001	0.11	0.01	0.34	0.047
Feeding × Age	0.91	0.65	0.93	0.75	0.66	0.49	0.43	0.27	0.25	0.86	0.22

Abbreviation: WOA, weeks of age.

a-d Means within a column and within a source with no common superscript differ (*P ≤* 0.05).

1 CON = control diet once a day; TAD = twice-a-day feeding: control diet twice a day; SF = split-feeding: special morning and afternoon diets.

**Figure 1:**
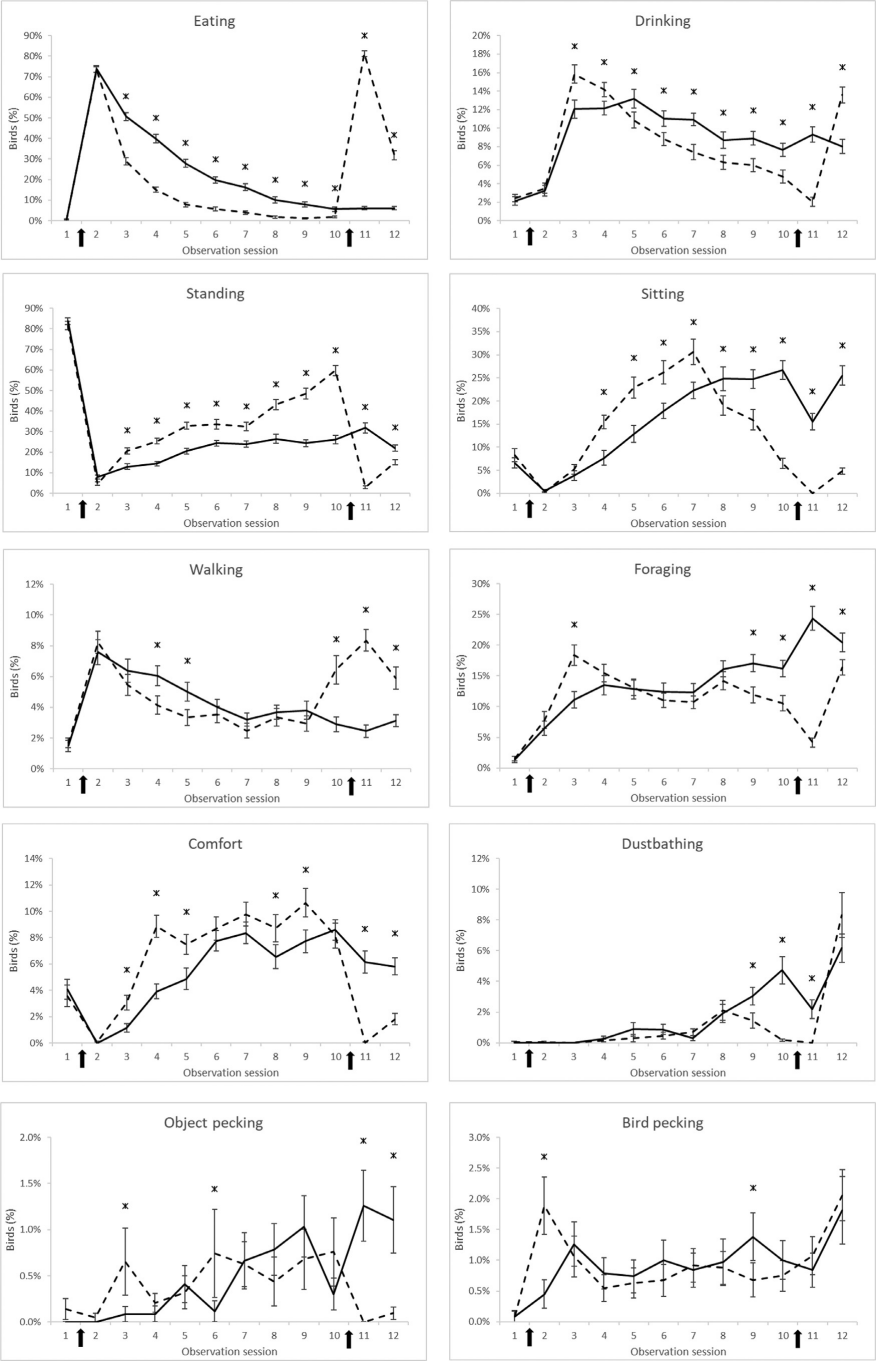
Effect of feeding strategies on the development of behavior over the 8 observation sessions. Arrows indicate the moment of feeding (first: 0730 h and second: 1600 h). Solid line = birds fed once a day and dashed line = birds fed twice a day. Error depicts the standard error of the mean (SEM). Asterisks indicate significant differences (*P* ≤ 0.05) between treatments.

Considering the behavior during the observation days, the TAD and SF treatments did not differ and were therefore combined as twice-a-day feeding against once-a-day feeding (CON). Significant interactions (*P* ≤ 0.05) between feeding frequency and observation sessions were found for all types of behavior (Figure 1). During the second observation session, after the first feeding moment (7:30 am), about 75% of the birds, on average, were eating. In the once-a-day fed birds, eating behavior decreased slowly during the day to approximately 5% between the 10th and 12th observation sessions. The birds fed twice a day showed faster decreased eating behavior and was around 2% between the 8th and 10th observation sessions. After the second feeding moment of the birds fed twice a day, more than 80% of the birds were eating, after which they decreased rapidly. These observations were in close agreement with those of van Emous and Mens (2021). Although the birds had access to water during the first observation session, only 2% of the birds were drinking, and after feed distribution, this was still low (3–4% of the birds) due to the high percentage of birds eating. The percentage of drinking birds was considerably higher during the third observation session. The birds fed twice a day showed more drinking during the third observation session and less drinking behavior after the fifth observation session compared to the birds fed once a day. This was caused by the lower amount of feed (50% of the daily portion) that the birds received in the morning compared to the birds fed once a day. During the last observation session, more birds fed twice a day were drinking. The drinking behavior pattern was comparable to a previous study from our lab (van Emous and Mens, 2021). Directly after lights on and before feeding, most of the birds fed once or twice a day (over 80%) stood. The birds fed twice a day showed increasing standing behavior during the day. It increased from observation sessions 2 to 10, from approximately 5 to 60% of the birds. After the second feeding, the percentage of birds standing was considerably lower. During the day, the birds fed once a day showed a slight increase in standing of about 10 to 30% after the first observation session. Just before the first feeding moment, approximately 8% of the birds sat on litter or slats. After the first feeding moment, hardly any birds showed sitting behavior. The percentage of birds fed twice a day had rapidly increased sitting behavior from approximately 0 to 30% between the second and seventh observation sessions. After that session, the percentage of birds sitting decreased rapidly, from approximately 30 to 0% between the 7th and 11th observation sessions. Sitting behavior in the once-a-day fed birds increased linearly to approximately 25% between the second and eighth observation sessions, after which it stabilized at approximately 25%, except for 1 dip during the 11th observation session. The resting behavior (standing and sitting) pattern was consistent with the previous research of van Emous and Mens (2021). Before the first feeding moment, the birds fed once and twice a day showed hardly any walking behavior, which was in contrast to expectations. In previous studies (e.g., Savory and Mann, 1997; de Jong et al., 2002), increased walking behavior (or pacing), specifically before feeding, was mentioned as an indicator of hunger or frustration from feed deficiency. In recent years, however, the conclusion that walking before feeding is indicative of hunger in broiler breeders has been questioned (Mens et al., 2022). In a study with pullets fed once or twice a day, the once-a-day fed pullets showed less walking or pacing behavior when those in adjacent pens received the second feed proportion. They postulated that due to habituation, birds know that they will be fed (specific sounds, biological clock, and the entrance of the caretaker). Due to this, the birds respond by becoming more active and walking up and down in anticipation of receiving feed. It is therefore questioned whether walking behavior before feeding is an indication of hunger or whether normal behavior corresponds to the expectation that birds will receive feed. Moreover, this specific behavior is also observed in captive wild birds under little or no feed restriction (McPhee and Carlstead, 2010).

After the first feeding moment, walking behavior decreased linearly for all treatments and stabilized for the once-a-day fed birds at around 3% between the 7th and 12th observation sessions. The birds fed twice a day showed increased walking behavior between the 10th and 12th observation sessions.

The birds fed twice a day showed more foraging at the third observation session and less foraging between the 9th and 12th observation sessions compared to the birds fed once a day. The birds fed twice a day showed more comfort behavior between the third and fifth observation sessions and at the ninth round, whereas after the second feeding moment, they showed less comfort behavior than the birds fed once a day. There was no difference in dustbathing behavior during the major part of the day. Only around the second feeding (between the 9th and 11th observation sessions) did the birds fed twice a day show less dustbathing behavior.

The birds fed twice a day showed less object pecking after the second feeding (11th and 12th observation sessions) than the birds fed once a day. The birds fed twice a day showed more pecking behavior toward other chickens during the second observation session.

Overall, birds fed twice a day showed more resting (standing and sitting) and comfort behavior between the 2 feeding times. This is an indications of a lower state of hunger and higher satiety (Hocking and Maxwell, 1996) resulting in better welfare.

## CONCLUSIONS

The present study showed that twice-a-day feeding with the same or different diets in the second phase of the production period affected production performance and behavior in broiler breeders. Compared to breeders fed the once a day control diet, split-fed breeders showed a tendency toward higher egg production between 45 and 65 WOA. The birds fed twice a day (TAD and SF) tended to have a lower water intake and water-to-feed ratio compared to the birds fed once a day. No differences were found for other production characteristics, egg weight, eggshell quality, fertility, feather cover, or embryonic mortality. The SF birds tended to have a 5% lower mortality rate than the TAD birds, whereas the CON birds did not differ from the other treatments. Due to twice a day feed distribution, the TAD and SF birds showed considerably different behavioral patterns than the CON birds. In conclusion, twice-a-day feeding decreases the water intake and water-to-feed ratio, whereas split-feeding tended to an improved egg production in broiler breeders. However, no effects were observed on eggshell quality and incubation traits, whereas the behavioral pattern of the twice-a-day fed birds was considerably different with potential better welfare.
